# Histological validation of a new CMR T1-mapping-based protocol to improve accuracy for fibrosis assessment in patients with aortic stenosis

**DOI:** 10.1186/1532-429X-18-S1-Q56

**Published:** 2016-01-27

**Authors:** Vassilis Vassiliou, Katharina Wassilew, George Asimakopoulos, Anthony de Souza, Cesare Quarto, Ee Ling Heng, Claire E Raphael, Bruce S Spottiswoode, Andreas Greiser, Evangelia Nyktari, Francisco Alpendurada, David Firmin, Andrew Jabbour, John Pepper, Dudley J Pennell, Peter Gatehouse, Sanjay Prasad

**Affiliations:** 1 grid.439338.6CMR, Royal Brompton Hospital, London, United Kingdom; 2 grid.7445.20000000121138111National Heart and Lung Institute, Imperial College London, London, United Kingdom; 3 Department of Cardiothoracic and Vascular Surgery, Deutsches Herzzentrum Berlin, Cardiac Pathology Unit, Berlin, Germany; 4 Siemens Healthcare, Erlangen, Germany; 5 grid.437825.f0000000091192677St Vincent's Hospital, Sydney, NSW Australia; 6 grid.439338.6Cardiothoracic Surgery, Royal Brompton Hospital, London, United Kingdom

## Background

Short 11-heart beat (11 HB) MOLLI have been proposed as a non-invasive method for the assessment of overall and diffuse fibrosis, but histological correlation has only been modest. We investigated an 11 HB MOLLI protocol with incremental acquisition of basal and mid-level images as a means for obtaining a global value of extracellular volume fraction (ECV). We validated this against intraoperative myocardial biopsies in patients with aortic stenosis.

## Methods

Ten patients (8 male, age 73 ± 7 years) with aortic stenosis (AS) scheduled for surgical valve replacement (3 with coronary artery disease) underwent CMR at 1.5T (Magnetom Avanto, Siemens Healthcare) with native 5(3)3 and post-gadolinium 4(1)3(1)2 T1 maps (Siemens prototype WIP 448B). Gadolinium 0.1 mmol/kg was administered, and post-gadolinium T1 maps were taken 15 later. The hematocrit was measured in an approved biochemistry laboratory on the same day. The MOLLI sequence was acquired twice at a single basal short-axis left-ventricular level, and twice at a single mid-ventricular level. Regions of interest were drawn in the septum at both levels to acquire T1 values. ECV calculation utilized an increasing number of maps as shown in table 1, using ECV=(1-hematocrit)* [(1/T1myocardium post contrast-1/T1 myocardium native)]/[(1/T1 blood post contrast-1/T1 blood native)].

**Figure 1:**
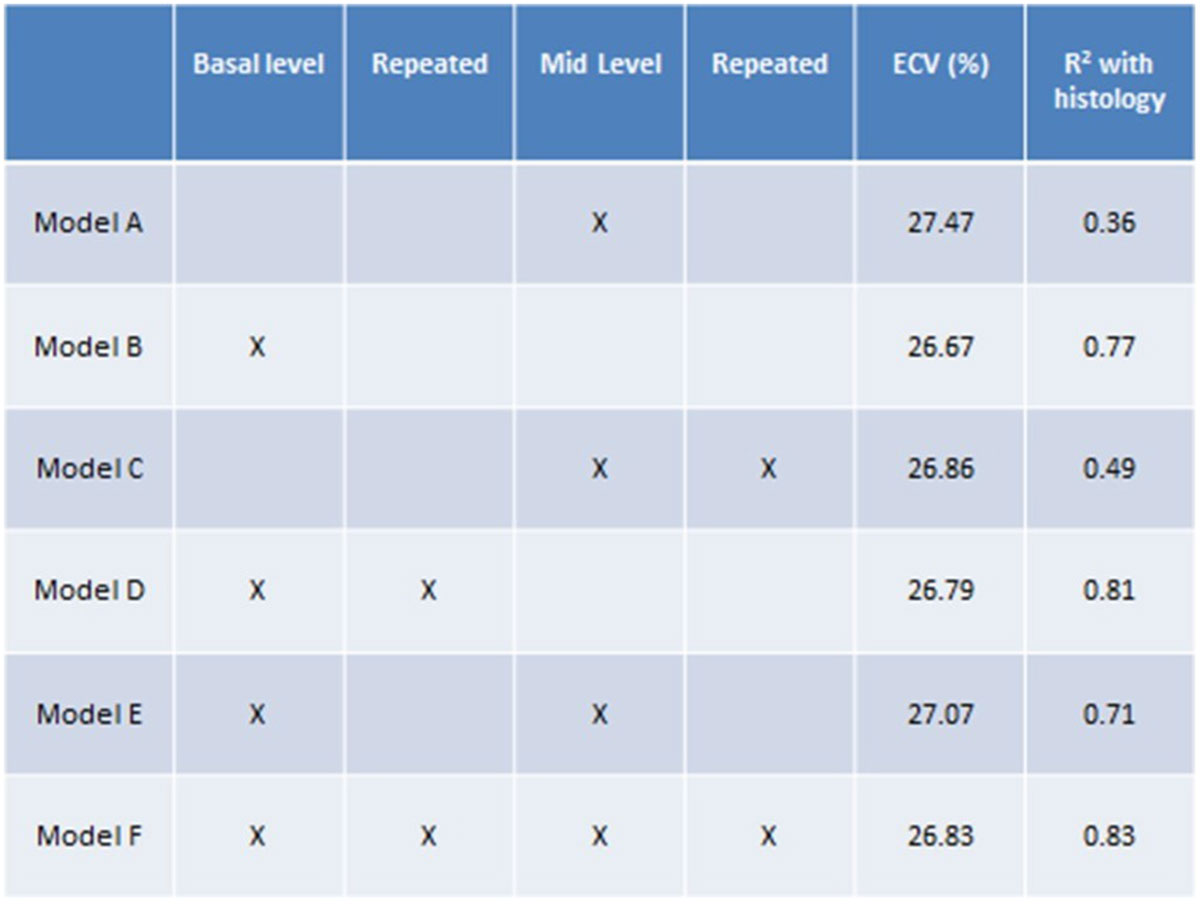
**Calculated ECV values using incremental MOLLI image acquisitions for each patient allowing convergence to the true global ECV**.

The following aspects of the methods are proposed as novel steps:

1. "Trucut" biopsy taken intraoperatively from the apical anterior/ lateral wall through the epicardium to allow histological characterization of the full myocardial wall.
2. A CMR imaging model including incrementally more acquisitions was applied as shown in Figure 1.

The myocardial biopsies were fixed in warm buffered formalin. Analysis of formalin-fixed paraffin-embedded, transmural myocardial biopsies of the left ventricle was performed on hematoxylin/eosin and Picrosirius Red-stained 3-micron-thick sections by a blinded experienced cardiac pathologist. Images were analysed using a purpose-built software to determine the extent of overall and reactive interstitial fibrosis.

## Results

**Figure 2:**
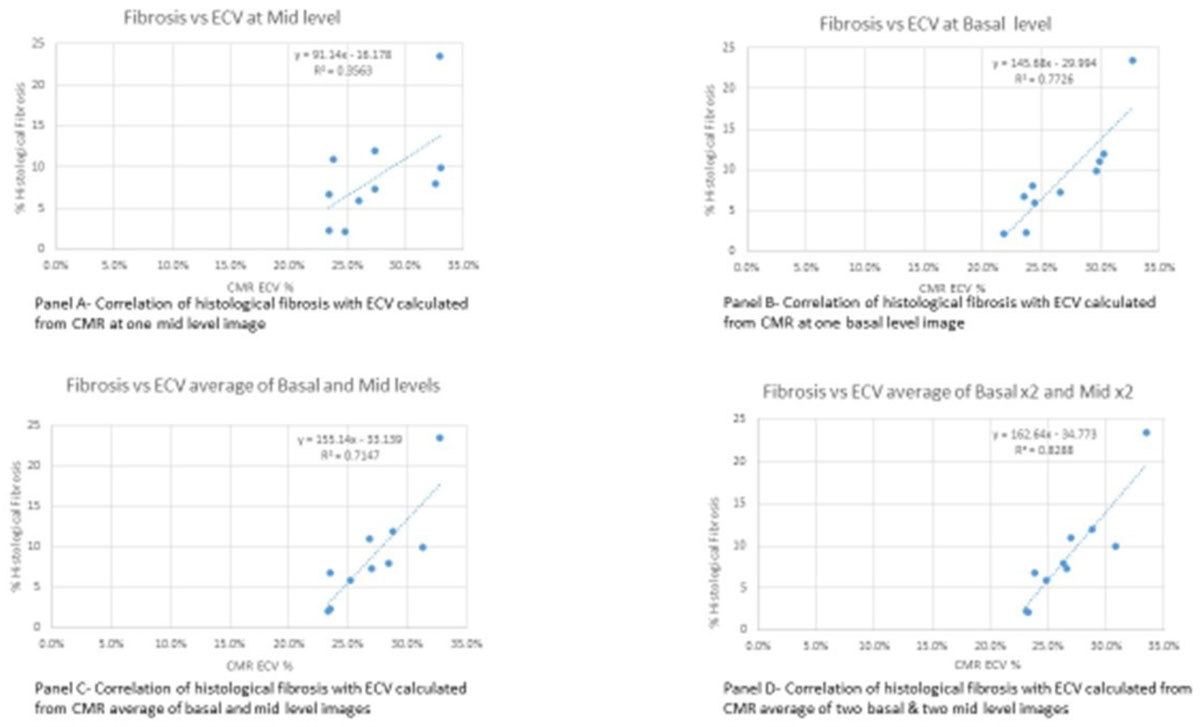
**Correlation between CMR-calculated ECV and histologically identified fibrosis**. The correlation proportionally increased as more levels and repeat sequences were included. Panel A top left: correlation with one mid-level, model A. Panel B top right: correlation with one basal level, model B. Panel C bottom left: correlation with average ECV obtained from the average of one basal and one mid-image, model E. Panel D bottom right: correlation with average ECV obtained from a total of two basal and two mid-level ECV, model F.

When only one mid-level MOLLI was taken correlation with histology was modest (R^2^ = 0.36; Figure 2, panel A, model A). Increasing the number of image acquisitions and including the basal level increased the correlation significantly: R^2^ = 0.76 when the average of a single basal and single mid-level image was used for ECV (C, model E) and importantly R^2^ = 0.83 when acquisition was repeated at both basal and mid-levels (panel D, model F).

## Conclusions

We have confirmed that ECV can provide accurate correlation with histological overall fibrosis. This work has demonstrated that the accuracy increases when MOLLI sequences are acquired at both basal and mid-ventricular levels, and further improves if repeated at each level. We conclude that an average T1 over 2 basal and 2 mid-ventricular level acquisitions improves correlation with histology in aortic stenosis patients when using 11 HB MOLLI.

